# Supplementary material for: Ingestion of Soybean Sprouts Containing a HASPIN Inhibitor Improves Condition in a Mouse Model of Alzheimer’s Disease
Source: Biology (Basel). 2023 Feb 16;12(2):320. doi: 10.3390/biology12020320 (PMC9953708; doi:10.3390/biology12020320)
Supplement: Supplementary file 1 [file biology-12-00320-s001.zip › biology-2217531-supplementary.pdf]

Table S1 Effects of diets on body weights**.**

Diets	Standard diet	Soybean sprouts*
Male	27.8±0.9	25.6±1.0 (0.0001)
Female	23.1±0.6	22.6±1.5 (0.73)

*Soybean sprouts means the diet including soybean sprout.

Six individuals were measured for each.

The numbers of in parentheses indicated *p* values in a standard diet and the diet including soybean sprout.
